# The Effects of Glutathione on Clinically Essential Fertility Parameters in a Bleomycin Etoposide Cisplatin Chemotherapy Model

**DOI:** 10.3390/life13030815

**Published:** 2023-03-17

**Authors:** Hale Bayram, Yaprak Donmez Cakil, Mustafa Erinc Sitar, Gamze Demirel, Belgin Selam, Mehmet Cincik

**Affiliations:** 1 Graduate School, Maltepe University, Istanbul 34857, Turkey; halebayram@maltepe.edu.tr; 2 Department of Histology and Embryology, Faculty of Medicine, Maltepe University, Istanbul 34857, Turkey; mehmet.cincik@maltepe.edu.tr; 3 Department of Clinical Biochemistry, Faculty of Medicine, Maltepe University, Istanbul 34857, Turkey; 4 Experimental Animals Research and Application Center, Maltepe University, Istanbul 34857, Turkey; 5 Cancer and Stem Cell Research Center, Maltepe University, Istanbul 34857, Turkey; gamzedemirel@maltepe.edu.tr; 6 Department of Obstetrics and Gynecology, School of Medicine, Acibadem Mehmet Ali Aydinlar University, Istanbul 34752, Turkey

**Keywords:** bleomycin, chemotherapy, cisplatin, DNA fragmentation, etoposide, glutathione, spermatozoa

## Abstract

Chemotherapeutic agents used in the treatment of testicular cancer cause damage to healthy tissues, including the testis. We investigated the effects of glutathione on sperm DNA integrity and testicular histomorphology in bleomycin etoposide cisplatin (BEP) treated rats. Twelve-week-old male rats of reproductive age (*n* = 24) were randomly divided into three groups, the (i) control group, (ii) BEP group, and (iii) BEP+ glutathione group. Weight gain increase and testes indices of the control group were found to be higher than that of the BEP group and BEP+ glutathione group. While the BEP treatment increased sperm DNA fragmentation and morphological abnormalities when compared to the control group, GSH treatment resulted in a marked decrease for both parameters. Moreover, BEP treatment significantly decreased serum testosterone levels and sperm counts in comparison to the control group, yet this reduction was recovered in the BEP+ glutathione treated group. Similarly, seminiferous tubule epithelial thicknesses and Johnsen scores in testicles were higher in the control and BEP+ glutathione groups than in the BEP-treated group. In conclusion, exogenous glutathione might prevent the deterioration of male reproductive functions by alleviating the detrimental effects of BEP treatment on sperm quality and testicular histomorphology.

## 1. Introduction

Testicular cancer (TC) is a malignancy frequently seen in men of reproductive age, with approximately 75,000 cases and over 9000 deaths per year worldwide. Though, in general, mortality rates have decreased or become stable due to the improvements in treatment, the incidence of TC has significantly increased in recent years in White males with the highest incidence in the Scandinavian countries, Germany, Switzerland, and New Zealand [1]. Generally, a favorable prognosis with a >90% cure rate and >95% five-year survival rate is achieved with effective management [2,3,4]. Cisplatin-based chemotherapy is the routine treatment of choice for patients who are at (i) early stages of nonseminomatous germ cell TC with risk factors, (ii) stage II seminoma, (iii) recurrence and (iv) advanced disease cases [5]. Three to four cycles of bleomycin, etoposide, and cisplatin (BEP) have become the treatment of choice in patients with metastatic germ cell tumors [6]. However, fertility becomes a major concern, as most patients with TC are of reproductive age and the BEP regimen is gonadotoxic [4].

The deleterious effects of BEP on semen parameters and sperm DNA persist even 24 months after the end of the treatment [7,8]. Specifically, cisplatin is associated with a high risk for prolonged or permanent infertility, with 20 to 47% of low versus high dose groups suffering from azoospermia after 5 years [9,10]. Moreover, 89% of patients were recorded to have elevated follicle-stimulating hormone (FSH) levels for 12 months after cisplatin-based chemotherapy including BEP, which was evident for more than 8 years in 64.3% of men, indicating persisting damage to Sertoli cell function [11]. Furthermore, BEP-treated patients with mild Leydig cell dysfunction during the initial standard 5-year follow-up period presented a significant long-term decline in age-adjusted testosterone levels, and hence a risk of testicular failure [12]. In accordance with the aforementioned perturbations, the paternity rate in long-term survivors of TC was found as 71%, and 6.6 years was the average duration from diagnosis until the birth of the first child [13]. A recent review, providing comprehensive information on sperm DNA damage in TC patients with clinical implications, reported a more common use of assisted reproductive technologies (ART), especially intracytoplasmic sperm injection (ICSI) by TC survivors, due to poor sperm quality when compared to the general population, with only 50% of couples achieving pregnancy. Maintenance of sperm chromatin integrity becomes specifically important in this issue due to the associated risk of transmitting defective genetic material to the embryo [14].

Sperm DNA fragmentation is the disruption of sperm chromatin integrity due to various intrinsic or extrinsic factors. Together with the abnormal chromatin condensation and dysregulation of normal apoptotic mechanisms, oxidative stress with the generation of reactive oxygen species (ROS) is among the primary causes of alterations in DNA integrity [15]. From a clinical perspective, the high level of sperm DNA damage is associated with a series of adverse reproductive outcomes [16].

Antioxidants possess the ability to reduce the deleterious effects of oxidative stress and protect the body against chemically induced toxicities [17,18]. Oral antioxidant treatments hold promise as adjuvants in chemotherapy [15,16,19]. Antioxidants neutralize ROS, which cause damage to cellular structures including DNA [20]. Several antioxidants such as aescin, coenzyme Q10, glutathione (GSH), L-carnitine, omega-3, selenium, zinc (Zn), and folate were shown to have positive effects on male fertility by mainly maintaining normal sperm motility and/or morphology [21,22]. A Cochrane review compared randomized controlled trials on the effectiveness of oral antioxidant supplementation in subfertile males, and demonstrated improved clinical pregnancy and live birth rates [23]. The 2019 review [24] included four studies evaluating sperm DNA fragmentation, and suggested a higher likelihood of achieving a pregnancy with decreased sperm DNA fragmentation. There is a recent update on the Cochrane review reporting similar reproductive outcomes in subfertile men, though more data is required to clarify the exact effects of the antioxidants [25]. 

Glutathione (GSH) is a tripeptide composed of glutamic acid, glycine, and cysteine. The latter provides the sulfhydryl group (–SH), which is involved in reduction and conjugation reactions, and makes GSH a major antioxidant synthesized in the cells [26]. Among its diverse functions, GSH is also involved in spermatogenesis and sperm maturation [27], and intracellular sperm GSH system components were reported to be linked to altered sperm morphology in infertile men [28]. Moreover, improvement of sperm quality was evident with exogenous GSH supplementation in infertile men with unilateral varicocele or genital tract inflammation [29], and in diabetic mice [30]. 

This study was designed to investigate for the first time the effects of GSH on BEP-caused reproductive toxicity by analyzing (i) sperm concentration and morphology; (ii) sperm DNA fragmentation; (iii) serum testosterone levels, and (iv) testicular histomorphology in experimental rat models that mimic the three cycle BEP treatment in humans.

## 2. Materials and Methods

### 2.1. Experimental Animals

Twenty-four Sprague-Dawley male rats, 12 weeks old, mature at reproductive age and produced in Maltepe University Experimental Animal Center, were used in the current study. Each cage contained a maximum of four rats, which were maintained at room temperature with a humidity of 50–60% and automatic 12-h light/dark cycle periods. All animals had free, and easy access to water and pellet food. The experimental protocol was applied in accordance with ethical approval given by Maltepe University Experimental Animal Local Ethics Committee (protocol number 2021.09.01).

### 2.2. BEP and GSH Treatment Protocols

Rats were randomly divided into three groups as i-control, ii-BEP and iii-BEP + GSH groups. The BEP protocol was applied for a total of 9 weeks, and the medication doses were determined in a preliminary study based on the study performed by Kilarkaje et al. [31]. However, 100% mortality was observed, and the dose of BEP treatment was reduced following subsequent experiments with lower doses. According to the results of the preliminary study, the treatment protocol included 0.17 mg/kg cisplatin (CAS: 15663-27-1; Koçakfarma, Istanbul, Turkey), 0.83 mg/kg etoposide (CAS: 33419-42-0; Koçakfarma), and 0.083 mg/kg bleomycin (CAS: 11056-06-7; Koçakfarma). 

The rats in the BEP group were treated with a 21-day 3-cycle protocol. Cisplatin and etoposide were administered on days 1–5, while bleomycin was given on days 2, 9 and 16 of each cycle by ip injection with 30-min intervals. The control group was injected with 0.9% NaCl, when bleomycin, etoposide, and cisplatin were applied to the BEP group. The BEP + GSH group was also administered with GSH (CAS: 70-18-8; Laboratorio Farmaceutico, Imperia, Italy) (200 mg/kg) twice a week by ip for 9 weeks in addition to the BEP treatment [32].

### 2.3. Tissue Removal and Sperm Collection

At the end of the experimental period, the rats were anesthetized by intraperitoneal administration of 100 mg/kg ketamine (Ketalar, CAS: 6740-88-1; Pfizer, Turkey) and 10 mg/kg xylazine (Rompun, CAS: 23076-35-9; Bayer, Turkey). All rats were sacrificed by taking intracardiac blood under general anesthesia, and their epididymis were removed and trimmed free of fat. Both testes and epididymis were rapidly excised and placed in a petri dish on ice. Their weights were recorded. Sperm was collected by mincing the epididymis in a global medium (LifeGlobal^®^ Media, Ballerup, Denmark) and incubating at 37 °C for 15 min.

### 2.4. Serum Testosterone Level Measurement

Intracardiac blood samples were obtained under full anesthesia and were placed in routine biochemistry tubes. Next, they were centrifuged at 1500× *g* for 15 min. In this way, serum was obtained and separated into aliquots. Testosterone levels were measured using chemiluminescence method with Siemens Immulite^®^ (Erlangen, Germany) device following the protocol recommended by the manufacturer. 

### 2.5. Semen Analysis

Sperm samples were diluted 10× with phosphate buffered saline (PBS), and 10 μL of the suspension was placed on a Makler counting chamber. The number of sperm heads was counted using a phase-contrast microscope under magnification of 20×. The sperm count was recorded to calculate the concentration of spermatozoa (10^6^/mL).

The Spermac stain method (FertiPRO, Beernem, Belgium) was used for sperm morphology evaluation according to the manufacturer’s guidelines. Semen smears were prepared and air-dried at 25 °C for 15–20 min. Slides were fixed in Spermac fixative solution and washed in distilled water. Subsequently, they were kept in Spermac A for 60 s, in Spermac B for 45 s, and in Spermac C for 45 s. In between, each dye was washed off with distilled water. The stained slides were air-dried and evaluated with a light microscope (Zeiss^®^, Oberkochen, Germany) under a magnification of 40×. At least 200 sperm were counted repetitively and evaluated for head, and tail abnormalities according to the Rat Sperm Morphological Evaluation Guide [33].

### 2.6. Sperm DNA Fragmentation Analysis

Sperm DNA fragmentation analysis was performed by the TUNEL (terminal deoxynucleotidyl transferase dUTP nick end labeling) method using the Abcam TUNEL assay kit-FITC (CAS: 3326-32-7; ab66108; Cambridge, UK). First, the sperm were fixed in 1% paraformaldehyde for 15 min at −20 °C. The fixed samples were centrifuged at 400× *g* for 7 min and the supernatants were removed. After washing twice in PBS, pellets were resuspended with 1 mL of ice-cold ethanol (70% *v*/*v*) until staining with the TUNEL assay kit according to the manufacturer’s instructions. The samples were analyzed for DNA fragmentation under a Zeiss LSM 700 confocal scanning microscope. Negative and positive samples of the kit were included as controls and at least 200 cells were counted for each sample. 

### 2.7. Testicular Histomorphology

Testicular tissues were fixed in 10% formaldehyde, embedded in paraffin blocks, and cut into sections of 2–3 μm thickness. Hematoxylin-eosin (H&E; CAS: 517-28-2, 6359-04-2) staining was performed for general histopathological evaluation at 10× and 40× magnification under a Zeiss light microscope by Johnsen scoring, which gives scores from 1 to 10 based on the stage of spermatogenesis (score of 10 corresponding to a maximum spermatogenesis activity, and score of 1 corresponding to a complete absence of germ cells) [34].

### 2.8. Statistical Analysis

The IBM SPSS Statistics 26 (Statistical Package for the Social Sciences; New York, NY, USA) program was used for statistical analysis. Kolmogorow-Smirnov and Shapiro-Wilk tests were used to determine whether the data conformed to a normal distribution. One-way ANOVA test was employed for three-group comparisons of normally distributed quantitative variables and Tukey post-hoc test was used for pairwise comparisons between the groups. The Kruskal-Wallis test and Tamhane’s post-hoc test were used in the comparison of three groups of quantitative variables that did not show normal distribution. Differences were considered statistically significant when *p* < 0.05. The bar graphs were created using GraphPad Prism V.8.01 (San Diego, CA, USA).

## 3. Results

### 3.1. Effects of BEP and BEP + GSH on the Weights of the Rats and the Reproductive Organs

The beginning and the final body weights (g), change in body weight (g), right and left testis weights (g), testes index (%), right and left epididymis weights (g), and epididymis index (%) of the control, BEP treated, and BEP + GSH treated rats, are shown in Table 1. BEP treatment significantly reduced the body weight gain (*p* = 0.027), right testis weight (*p* = 0.002), left testis weight (*p* = 0.027), testes index (*p* = 0.017), right epididymis weight (*p* = 0.002), and left epididymis weight (*p* = 0.010), and the epididymis index (*p* = 0.000) in comparison to the control group. Similar decreases were also obtained in BEP + GSH group when compared to the control group (*p* = 0.012 for body weight gain, *p* = 0.000 for right testis weight, *p* = 0.000 for left testis weight, *p* = 0.001 for testes index, *p* = 0.000 for right epididymis weight, *p* = 0.004 for left epididymis weight, and *p* = 0.000 for epididymis index). No significant differences were found in the respective parameters when the BEP and BEP + GSH groups were compared (*p* > 0.05).

### 3.2. Effects of BEP and BEP + GSH Treatments on Sperm Count and Morphology

**Figure 1:**
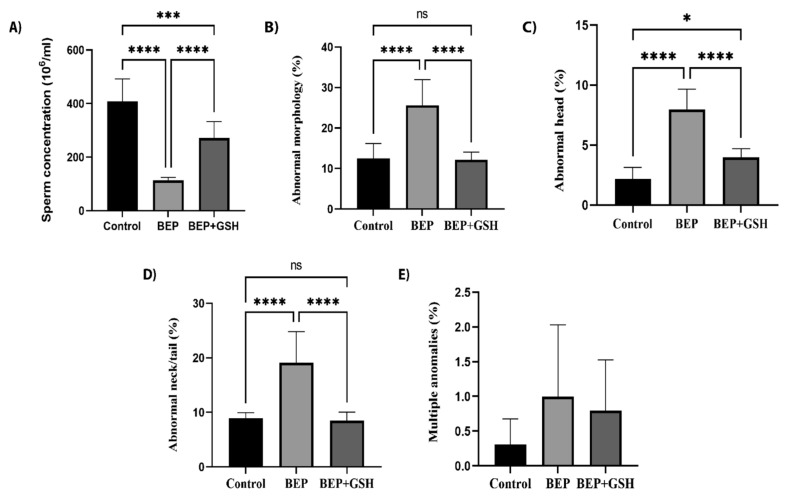
Effects of GSH on sperm parameters at the end of the experimental period. Control, BEP treated and BEP + GSH treated groups were compared for (**A**) sperm concentration (10^6^/mL), (**B**) abnormal morphology (%), (**C**) abnormal head (%), (**D**) abnormal neck/tail (%), and (**E**) multiple abnormalities. Data are expressed as mean ± SD. One-way ANOVA was used to assess the significance among the groups of (**A**–**D**), while the Kruskal Wallis test was used for the statistical evaluation of E. ns: not significant, * *p* < 0.05; *** *p* < 0.001; **** *p* < 0.0001.

Sperm count analysis and sperm morphology evaluation were performed to assess the effects of BEP and BEP + GSH treatments on spermatogenesis (Figure 1). As shown in Figure 1A, BEP treatment caused a marked decrease in sperm concentration (10^6^/mL) in comparison to the control group (112.5 ± 4.1 vs. 408.1 ± 29.6, *p* = 0.000), which was partially recovered in BEP + GSH group (271.2 ± 21.8, *p* = 0.001 in comparison to BEP group). Similarly, while the percentage of the sperm morphological abnormalities (Figure 1B) increased dramatically in BEP group (25.6 ± 2.27 vs. 12.5 ± 1.3 in the control group, *p* = 0.000), a drastic decrease was observed when GSH is administered (12.1 ± 0.7, *p* = 0.000 in comparison to BEP group). 

**Figure 2:**
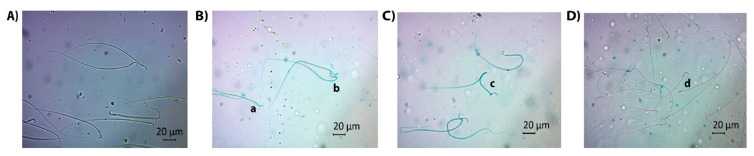
Light microscopic images for illustrating various morphological abnormalities: (**A**) normal sperm, (**B**) a: flattened head, b: bent neck, (**C**) c: multiple abnormalities (bent neck and tail), (**D**) d: pinhead sperm.

Morphological abnormalities were evaluated as abnormal neck/tail, abnormal head and multiple abnormalities, and the former had the highest incidence. Higher percentage of spermatozoa with abnormal head (Figure 1C) or abnormal neck/tail (Figure 1D) were obtained in the BEP group in comparison to the control group (*p* = 0.000 and *p* = 0.000, respectively). A significant decrease was observed in both parameters when GSH was administered (*p* = 0.000 and *p* = 0.000, respectively, when compared to the BEP group). The sperm counts with multiple abnormalities (Figure 1E) were similar among the study groups (*p* > 0.05). Figure 2 shows the images illustrating morphologically normal sperm and various morphological abnormalities.

### 3.3. Effects of BEP and BEP + GSH on Sperm DNA Fragmentation

**Figure 3:**
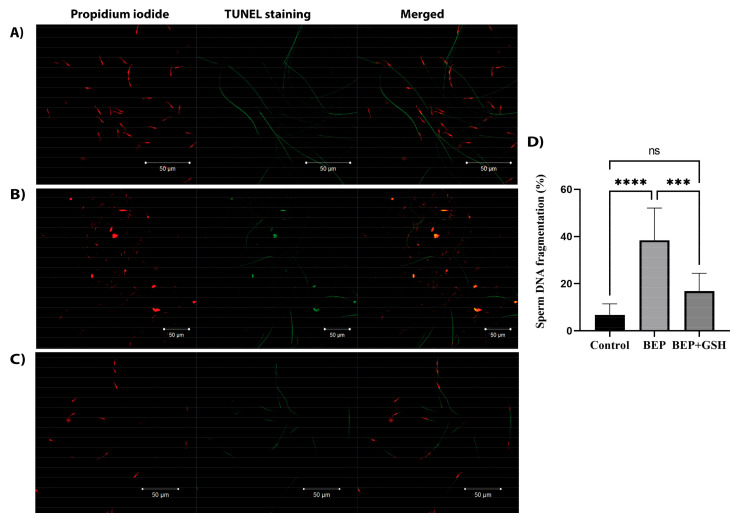
Representative immunofluorescence images showing sperm DNA fragmentation in (**A**) control, (**B**) BEP-treated, and (**C**) BEP + GSH-treated groups. Propidium iodide staining in the left panel, TUNEL staining in the middle panel and merged images in the right panel. In the merged images, sperm heads with red fluorescence correspond to intact DNA, while sperm heads with yellow to orange fluorescence indicate fragmented DNA. (**D**) Bar graph demonstrating the significant differences among the groups. One-way ANOVA test was performed for statistical evaluation of significance, and the data are presented as mean ± SD. *** *p* < 0.001; **** *p* < 0.0001.

The TUNEL immunofluorescence assay was performed to investigate DNA integrity in control, BEP and BEP + GSH groups (Figure 3). Sperm heads with intact DNA were only stained with propidium iodide (PI) and appear red in the merged images in the right panel. On the other hand, TUNEL positive sperm heads were both stained with TUNEL stain and PI, and displayed yellow to orange fluorescence in the merged images (Figure 3A–C). Sperm DNA fragmentation was dramatically higher in the BEP group compared to the control and BEP + GSH groups (6.8 ± 1.6% vs. 38.4 ± 4.8% for control vs. BEP groups, *p* = 0.000; 38.4 ± 4.8% vs. 16.9 ± 2.7% for BEP vs. BEP + GSH groups, *p* = 0.000). There was no statistically significant difference between the control and the BEP + GSH groups (6.8 ± 1.6% vs. 16.9 ± 2.7%, *p* = 0.105) (Figure 3D).

### 3.4. Effects of BEP and BEP + GSH on Serum Testosterone Levels

**Figure 4:**
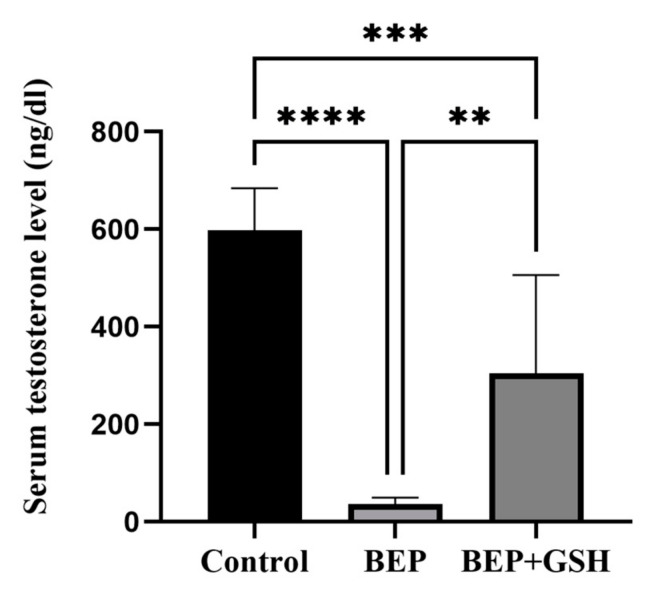
Effects of GSH on serum testosterone levels (ng/dL) at the end of the experimental period. Data are given as mean ± SD. The differences among control, BEP treated and BEP + GSH treated groups were evaluated using one way-ANOVA test. ** *p* < 0.01; *** *p* < 0.001; **** *p* < 0.0001.

Serum testosterone levels were measured in control, BEP and BEP + GSH groups to examine the effects of BEP and BEP + GSH treatments on Leydig cell function (Figure 4). The results showed a significant decrease in serum testosterone following BEP treatment when compared to the saline treated control group (598.1 ± 30.5 vs. 36.1 ± 4.8; *p* = 0.000). However, when GSH was also administered, significantly increased testosterone levels were obtained in the BEP + GSH group in comparison to the BEP group (304.6 ± 71.4 vs. 36.1 ± 4.8; *p* = 0.001).

### 3.5. Effects of BEP and BEP + GSH on Testis Histomorphology

**Figure 5:**
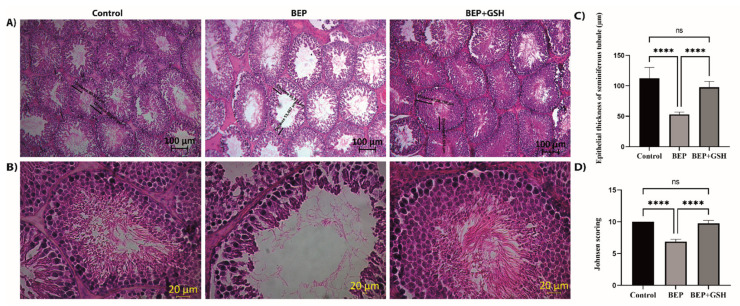
Paraffin-embedded testis tissue blocks were cut into sections of 2–3 μm thickness and stained with hematoxylin&eosin to demonstrate the histopathological changes in control, BEP treated and, BEP + GSH treated groups. Representative images under (**A**) 10×, (**B**) 40× magnification. Bar graphs demonstrating (**C**) epithelial thickness of the seminiferous tubules (μm) measured from 10 randomly selected areas of each group, and (**D**) the mean Johnsen testicular biopsy score. The differences among control, BEP treated and BEP + GSH treated groups were evaluated using one way-ANOVA test. **** *p* < 0.0001.

Testicular sections of 2–3 µm thickness were stained with H&E to examine if BEP treatment was associated with histopathological changes and, if any, these changes could be recovered by GSH treatment (Figure 5A,B). While spermatogenic cellular masses were observed in the lumen of seminiferous tubules in both control and BEP + GSH groups, the seminiferous tubule lumens were less occupied in the BEP group. 

To investigate the changes in a qualitative manner, the thickness of the seminiferous tubule epithelium (germinal epithelium height) (Figure 5C), and mean Johnsen testicular biopsy scores (Figure 5D) were calculated for each group. A marked decrease in the thickness of the seminiferous tubule epithelium was evident in BEP group in comparison to the control group (53.2 ± 1.3 vs. 112.2 ± 6.4, *p* = 0.000), which was reversed with administration of GSH in BEP + GSH group (53.2 ± 1.3 vs. 97.9 ± 3.3, *p* = 0.000). No significant difference was found when the control and BEP + GSH groups were compared (*p* > 0.05) (Figure 5C). A similar trend was observed when Johnsen scores were compared. The Johnsen score of the BEP group was lower than those of the control and BEP + GSH groups (10.00 ± 0.0 vs. 6.9 ± 0.1, *p* = 0.000, when control and BEP groups were compared; 6.9 ± 0.1 vs. 9.8 ± 0.2, *p* = 0.000, when BEP and BEP + GSH groups were compared). There was no significant difference between the control and the BEP + GSH groups (*p* > 0.05) (Figure 5D).

## 4. Discussion

Testicular tumors have great sensitivity to cisplatin-based chemotherapy including BEP, mainly due to insufficient DNA damage repair and hypersensitive apoptotic response to DNA damage [35]. At the same time, oxidative stress induces a reduction in sperm motility and damage to sperm DNA and is considered one of the main pathophysiological mechanisms of male infertility after treatment with these alkylating agents [36]. 

Physiologically, oxidative stress seems to contribute positively to acrosome reaction, hyperactivation, oocyte interactions, motility and capacitation of spermatozoa in males. This situation is actually like a double-edged sword [37]. Unfortunately, sperm cells are extremely sensitive to high ROS levels due to the oxidative peroxidation of unsaturated fatty acids, which are found in large amounts in their membranes. Additionally, cytoplasmic defense mechanisms are not present, which, together with the former, result in increased oxidative stress leading to the oxidation of sperm cell DNA, proteins, and lipids, subsequently altering sperm vitality, motility, and morphology [38]. Accumulating evidence points to a relationship between male subfertility and oxidative stress in variable cases [39]. 

Antioxidant supplementation is gaining increasing attention by alleviating chemotherapy-induced reproductive dysfunction in medical communities [16,19]. So far, several studies have demonstrated the protective effects of some antioxidant compounds such as melatonin, zinc, selenium, and α-tocopherol on the reproductive toxicity caused by BEP treatment [31,40,41]. Significant improvements in sperm count, motility, viability, morphology, testosterone levels, histopathology, and stereology of testes were reported in melatonin administered groups receiving BEP treatment [41]. Supplementation of zinc following BEP treatment restored chromatin integrity, testicular organization and spermatogenesis [40]. An antioxidant cocktail including α-tocopherol, L-ascorbic acid, zinc, and selenium was shown to protect testicular and reproductive endocrine functions when administered in conjunction with BEP therapy, and subsequently enhanced the recovery of BEP-induced testicular dysfunction [31]. Royal jelly also had positive effects on sperm count, viability, motility, and DNA integrity, and testosterone concentration in bleomycin-treated rats [42]. Recently, Abdel-Latif et al. reported that antioxidant kinedine used together with cisplatin prevented histopathological lesions in the testis, increased serum testosterone level and improved sperm motility [43]. 

Cisplatin-based chemotherapies are associated with reduced weights of reproductive organs, lower sperm count and motility, more frequent morphological abnormalities, and impairment in testicular histology [43,44,45]. A possible reason for lower testes weight is that the secretory and synthesis capacities decrease secondary to the decrease in mitotic activity with chemotherapy. In agreement with the findings of the current study, lower sperm counts, decreased testosterone secretion, increased sperm DNA damage, and altered testis histomorphology can be observed following BEP treatment [7,8,41,46]. Decreased testosterone levels can be observed when the equilibrium redox is lost [47]. The increase in DNA fragmentation, which is associated with many reproduction-related disease states, is undoubtedly related to infertility and subfertility. Ghezzi et al. reported significantly higher aneuploidy and DNA fragmentation following BEP treatment [7]. As anticipated, oxidative damage is the primary factor in increased DNA fragmentation in sperm cells [48]. Oxygen-derived free radicals can easily destabilize DNA and lead to fragmentation [49]. Oxidative stress and DNA fragmentation intersect at a clinical point as the high level of sperm DNA damage is associated with a series of adverse reproductive outcomes. Histological alterations following BEP chemotherapy in the testes are thought to be due to oxidant/antioxidant imbalances [41]. BEP treatment was shown to trigger oxidative stress and apoptosis of spermatogonial stem cells and Sertoli cells resulting in impaired spermatogenesis [50].

The testes mainly use enzymatic antioxidants such as copper/zinc superoxide dismutase and selenoenzyme phospholipid hydroperoxide glutathione peroxidase, as well as non-enzymatic antioxidants such as reduced GSH, to avoid the harmful effects of ROS [47,51,52]. GSH, considered one of the most important antioxidants, contributes to antioxidant defense both as a substrate for glutathione peroxidase and by directly sequencing free radicals [53]. Our experimental research aimed to ameliorate the chemotherapy-induced imbalance in redox hemostasis with exogenous systemic GSH supplementation. 

Systemic GSH application together with the BEP treatment prevented the deterioration of spermatogenesis and preserved the histological structure of the testis, as shown by the improved testes and epididymis indices, testosterone levels, histomorphological examinations, and reduced DNA fragmentation in comparison to BEP treatment alone. In parallel with these findings, sperm count was higher and morphological abnormalities were lesser in the GSH administrated group compared to the BEP-only-treated group. In terms of testicular histology, GSH restored Johnsen scores, and mean seminiferous tubule epithelial thicknesses in rats receiving BEP treatment. The molecular mechanisms of the positive effects of GSH in this study are suggested as (i) quenching of H_2_O_2_, (ii) preventing lipid peroxidation, (iii) stopping membrane oxidation, and (iv) contributing to the enzymatic redox cycle [54].

In agreement with our results, in another study, GSH deficiency was reported to lead to the instability of the spermatozoa midpiece, resulting in defective morphology and reduced motility of the spermatozoa [37]. In a study with diabetic mice, sperm motility was found to be higher when the mice were administered GSH [30]. In a recent study evaluating the possible protective effect of earthworm methanolic extract (EE) on impaired reproductive functions caused by acrylamide (ACR) toxicity, ACR application was shown to reduce testicular GSH, and decrease the sperm count, motility, and viability. On the other hand, the application of EE in combination with ACR protected testicular GSH levels [55]. Although there are differences regarding GSH concentration, these studies support evidence on the beneficial effects of GSH on protection from reproductive toxicity.

## 5. Conclusions

Exogenous supplementation of glutathione contributes positively to oxidative stress challenges in different biological systems such as cell cultures, plants, and animals at organismal levels [56,57]. This study revealed the beneficial effects of GSH on the restoration of testicular function, spermatogenesis, and sperm DNA integrity in BEP treated rats, possibly by reversing the deleterious effects of oxidative stress. Though our findings might suggest GSH supplementation as an adjuvant in BEP chemotherapy for alleviating reproductive toxicity, further in vivo studies are required to examine tumor response and therapeutic efficiency for potential clinical implications.

## Figures and Tables

**Table 1:** Starting and final body weights (g), change in body weight (g), testis weights (left and right, g), testes index (%), epididymis weights (left and right, g), and epididymis index (%) of control, bleomycin, etoposide, and cisplatin (BEP) treated, and BEP + glutathione (GSH) treated rats. Data are presented as mean ± SD. * significant difference compared to the control group (*p* < 0.05), ** significant difference compared to the control group (*p* < 0.001).

	Control	BEP	BEP + GSH
Beginning body weight (g)	323.5 ± 15.9	328.1 ± 11.9	338.13 ± 13.3
Final body weight (g)	398.0 ± 17.2	374.50 ± 11.8	380.6 ± 12.8
Change in body weight (g)	74.5 ± 10.6	46.4 ± 5.6 *	49.1. ± 2.8 *
Right testicular weight (g)	1.9 ± 0.06	1.6 ± 0.1 *	1.4 ± 0.05 **
Left testicular weight (g)	1.9 ± 0.6	1.64 ± 0.10 *	1.47 ± 0.058 *
Testes index ^a^	0.9 ± 0.03	0.8 ± 0.05 *	0.8 ± 0.02 *
Right epididymis weight (g)	0.8 ± 0.05	0.6 ± 0.05 *	0.573 ± 0.02 **
Left epididymis weight (g)	0.8 ± 0.07	0.6 ± 0.04 *	0.6 ± 0.02 *
Epididymis index ^b^	0.4 ± 0.02	0.33 ± 0.01 *	0.30 ± 0.01 *

^a^ Testis index = [(right + left testis weights)/body weight] × 100. ^b^ Epididymis index = [(right + left epididymis weights)/body weight] × 100.

## Data Availability

Not applicable.
